# Nothing About Us Without Us: Principles for Lived Experience‐Informed Psycho‐Oncology Education and Training

**DOI:** 10.1111/hex.70898

**Published:** 2026-10-07

**Authors:** Darren Haywood, Sue Woodall, Annika Hegde, Jai Carmichael, Evan Dauer, Alexia Samiotis, Nicolas H. Hart

**Affiliations:** ^1^ Human Performance Research Centre, School of Human Performance, Rehabilitation and Population Health, INSIGHT Research Institute, Faculty of Health University of Technology Sydney (UTS) Sydney New South Wales Australia; ^2^ Department of Mental Health St. Vincent's Hospital Melbourne Fitzroy Victoria Australia; ^3^ School of Population Health, Faculty of Health Sciences Curtin University Bentley Western Australia Australia; ^4^ Centre for Mental Health and Brain Sciences Swinburne University of Technology Hawthorn Victoria Australia; ^5^ LiveWorkCancer Sydney New South Wales Australia; ^6^ School of Psychological Sciences Monash University Clayton Victoria Australia; ^7^ Caring Futures Institute, College of Nursing and Health Sciences Flinders University Adelaide South Australia Australia; ^8^ Exercise Medicine Research Institute, School of Medical and Health Sciences Edith Cowan University Perth Western Australia Australia; ^9^ Cancer and Palliative Care Outcomes Centre, Faculty of Health Queensland University of Technology (QUT) Brisbane Queensland Australia; ^10^ Institute for Health Research University of Notre Dame Australia Perth Western Australia Australia

**Keywords:** co‐design, consumer involvement, health professional education, lived experience, patient partnership, psycho‐oncology, workforce development

## Abstract

Lived experience is increasingly recognised as an essential source of expertise in healthcare research, service design, policy and quality improvement. In psycho‐oncology, consumer involvement is now widely encouraged in research and service development; however comparatively little attention has been paid to the role of lived experience in educating and training the psycho‐oncology workforce. We argue that involving people with lived experience should become a minimum requirement in developing, delivering, and evaluating psycho‐oncology education and training. Given that psycho‐oncology is fundamentally concerned with understanding and responding to the lived experience of cancer consumer involvement in education and training must be expected. To support meaningful implementation, we provide Principles for Lived Experience‐Informed Psycho‐Oncology Education and Training: representation, partnership, support, and accountability. Embedding lived experience expertise throughout psycho‐oncology workforce development will strengthen the relevance, authenticity, and person‐centredness of future education and training initiatives and may improve consumer outcomes.

## 1 Introduction

1.1

Lived experience involvement has become increasingly prominent within contemporary healthcare research, policy, service design, and quality improvement. Across healthcare systems, there has been significant progress toward consumers no longer being viewed solely as recipients of care, but rather as partners in care, whose expertise provides unique insights into healthcare priorities, outcomes, and experiences [1, 2, 3]. This shift reflects growing recognition that professional and academic knowledge of a given health discipline or condition, while essential, does not fully capture the realities of navigating life and the health system when living with or beyond illness.

Psycho‐oncology has embraced many aspects of this necessary and valuable movement. Consumers and advocates are commonly involved in research priority setting, service evaluation, supportive care programme development, and clinical guideline development. This aligns with the fundamental aim of psycho‐oncology: understanding and responding to the psychosocial experiences of people affected by cancer. Yet, despite substantial progress in these domains, there is one area that remains comparatively underdeveloped; lived experience involvement within the development and delivery of psycho‐oncology education and training.

Over the past decade, psycho‐oncology workforce development has expanded considerably. Education and training initiatives include courses and modules on distress screening, communication skills, managing fear of cancer recurrence and progression, cognitive symptom management, sexuality, body image concerns, anxiety, depression, post‐traumatic stress, bereavement, grief, and end‐of‐life care. However, these programmes continue to be predominantly designed, delivered, and evaluated by clinicians, researchers, educators, and professional organisations. While many programmes aim to improve person‐centred practice, relatively few report meaningful involvement of people with lived experience in determining what clinicians should learn, how education and training should be delivered, or whether education and training objectives align with consumer needs. This omission raises an important question: If psycho‐oncology seeks to improve the experiences of people affected by cancer, shouldn't those same people help shape how the workforce is educated and trained? We argue that the next evolution of psycho‐oncology education and training should move beyond acknowledging the value of lived experience, and instead establish lived experience involvement as a minimum standard in clinician education and training.

## 2 Why Lived Experience Matters in Psycho‐Oncology Education and Training

Cancer can affect every aspect of life. Beyond physical symptoms and treatment toxicities, individuals must navigate uncertainty, identity disruption, traumatic experiences, altered relationships, work‐related challenges, financial burden, existential concerns, and fears regarding recurrence or progression. While psycho‐oncology clinicians are trained to address these issues, no amount of professional education can fully replicate the experiential knowledge held by those with lived experience of cancer.

Lived experience offers a perspective that differs fundamentally from professional expertise. Clinicians often develop conceptual understandings of distress, coping, adjustment and supportive care needs, while consumers provide direct insights into how these experiences unfold in everyday life, which issues matter most at different stages of the cancer journey, why they matter, and what forms of support are perceived as helpful, irrelevant, or even harmful. Consumer partnership in professional education and training is increasingly framed as learning from and with consumers, rather than learning about consumers [1]. Indeed, the value of explicit consumer involvement in health professional education and training is increasingly recognised across healthcare disciplines, ranging from consumer storytelling through to curriculum design, institutional governance, and learner assessment, arguing that higher levels of engagement provide greater opportunities for partnership and shared decision‐making—with meaningful consumer partnership requiring relational investment, ethical considerations, and organisational support [1, 4].

The rationale for consumer involvement may be particularly compelling in psycho‐oncology. Unlike many healthcare disciplines that focus primarily on disease processes, diagnostics, treatments, or technical competencies; psycho‐oncology is centrally concerned with understanding and responding to the human experience of cancer. The discipline seeks to improve communication, adjustment, coping, decision‐making, relationships, quality of life, and supportive care outcomes and experiences across the cancer continuum. These are inherently subjective phenomena that cannot be fully understood through clinical expertise alone. This is particularly evident in the skills that form the core components of psycho‐oncology practice. Psycho‐oncology clinicians are often involved in helping consumers navigate some of the most emotionally significant moments of the cancer journey, including receiving a diagnosis, discussing treatment options, managing uncertainty, coping with recurrence, transitioning to survivorship, and engaging in conversations about disease progression or end‐of‐life care. While clinicians can be taught evidence‐based frameworks, consumers are uniquely positioned to explain how information is received during these vulnerable moments, which language feels supportive or distressing, and how timing and delivery influence understanding. Consumers also offer direct insight regarding how care can foster or erode a person's sense of autonomy and control, what enables meaningful participation in decision‐making, how trust and therapeutic relationships are established, strengthened and maintained, what clinicians can do to prevent misunderstandings, strengthen engagement, and reduce therapeutic ruptures, and what helps people feel understood, empowered, and supported to navigate uncertainty, and what consumers need most from healthcare professionals at these times.

Ultimately, lived experience should not be viewed as a supplementary perspective in psycho‐oncology education and training, but as a form of expertise directly relevant to the discipline's core purpose. Excluding lived experience from psycho‐oncology education and training risks creating a disconnect between what clinicians are taught and how people experience cancer, navigate uncertainty, and exercise autonomy in their care—potentially contributing to gaps between evidence‐based care, consumer priorities, and clinical practice.

## 3 Lived Experience as Expertise

When lived experience is included in healthcare education and training involvement typically occurs through guest presentations or personal testimonials [4]. While these approaches can be powerful, they represent only one form of contribution, which are often single events rather than routine or enduring involvement. Limiting consumer involvement to sharing their own personal stories risks positioning lived experience primarily as educational content rather than as educational partners. This is a limited model of involvement, particularly when the goal is to prepare clinicians to understand and respond to the psychosocial complexities of cancer care. By contrast, meaningful partnership involves shared influence over educational design, delivery, evaluation, and governance. This distinction reflects broader developments within consumer engagement and co‐design frameworks, which increasingly emphasise partnership, reciprocity, and shared decision‐making, rather than consultation alone [5].

In psycho‐oncology education and training, lived experience contributors could be involved in identifying learning priorities, co‐developing curricula structure, reviewing educational materials, co‐facilitating training sessions, developing case studies, participating in competency assessment, interpreting evaluation findings, and informing continuous quality improvement processes. Health professional education examples demonstrate that consumers can successfully be involved in course development and implementation [1, 4]. Consumers can frequently identify priorities, experiences, and perspectives that may not be fully captured through professional or academic expertise. Consumer expertise can therefore influence not only what is taught, but also how future professionals understand and respond to psychosocial care needs [1, 4].

## 4 The Risk of Tokenism

Calls for greater consumer involvement in education and training must be accompanied by careful consideration of implementation. Not all involvement is meaningful, and poorly designed engagement risks tokenism. Tokenistic approaches typically occur when consumers are invited into processes with little influence over decisions, unclear expectations, inadequate preparation, or insufficient support. Such practices may satisfy organisational expectations regarding engagement while offering limited opportunity for genuine partnership. In health professional education and training, consumer partnership is most meaningful when consumers are recognised as people to learn from and with, rather than as symbolic participants in educator‐led activities [1, 2].

The consequences extend beyond ineffective education and training. While meaningful involvement can be personally rewarding and empowering for many contributors, tokenistic involvement may place emotional burdens on contributors who are repeatedly asked to share personal experiences without receiving adequate recognition, support, or compensation. Recounting cancer experiences may evoke distress, particularly when discussions involve diagnosis, treatment, recurrence, bereavement, or adverse healthcare experiences. Further, tokenism may unintentionally reinforce inequities in representation. Educational programmes often rely on highly engaged advocates who are accessible, experienced, and confident in navigating healthcare systems. While these contributors provide valuable perspectives, they cannot represent the diversity of experiences across cancer populations. Without deliberate efforts to broaden participation, educational and training design may fail to reflect the experiences of younger adults, older adults, culturally and linguistically diverse communities, Indigenous peoples, rural and remote populations, carers, LGBTIQA+ individuals, and people living with advanced or metastatic disease. These experiences are also shaped by intersecting factors such as socioeconomic circumstances, disability, comorbid health conditions, educational background, and caring responsibilities. Evidence suggests even when diverse consumer experiences are sought in co‐design initiatives in oncology, representation can remain limited by factors such as socioeconomic background and prior educational access [1, 5]. Addressing these barriers may require flexible and accessible approaches to involvement, such as online participation options, interpreters, culturally responsive engagement strategies, reimbursement of costs, and accommodations for disability, treatment demands, and caring responsibilities. Meaningful involvement therefore requires structured approaches that recognise lived experience as expertise rather than treating participation as voluntary advocacy alone.

## 5 Principles for Lived Experience‐Informed Psycho‐Oncology Education and Training

To advance the field, we propose four core principles to guide lived experience‐informed psycho‐oncology education and training: representation, partnership, support, and accountability. These principles are underpinned by key questions for psycho‐oncology educators to consider when developing education and training. The principles and guiding questions are informed by international guidelines and resources for consumer involvement in research and service development, evidence syntheses of consumer involvement in the education and training of health professionals, and the education, clinical, research, and lived experience expertise of our authorship group [1, 2, 3, 4, 5]. While the overarching principles may be broadly applicable across healthcare education, the principle descriptions and guiding questions have been specifically tailored to inform psycho‐oncology education and training initiatives. They reflect the importance of diverse cancer‐related experience, psychosocial support, and understanding how people experience and respond to cancer across the cancer journey. A graphical depiction of the principles and guiding questions is provided in Figure 1.

**Figure 1:**
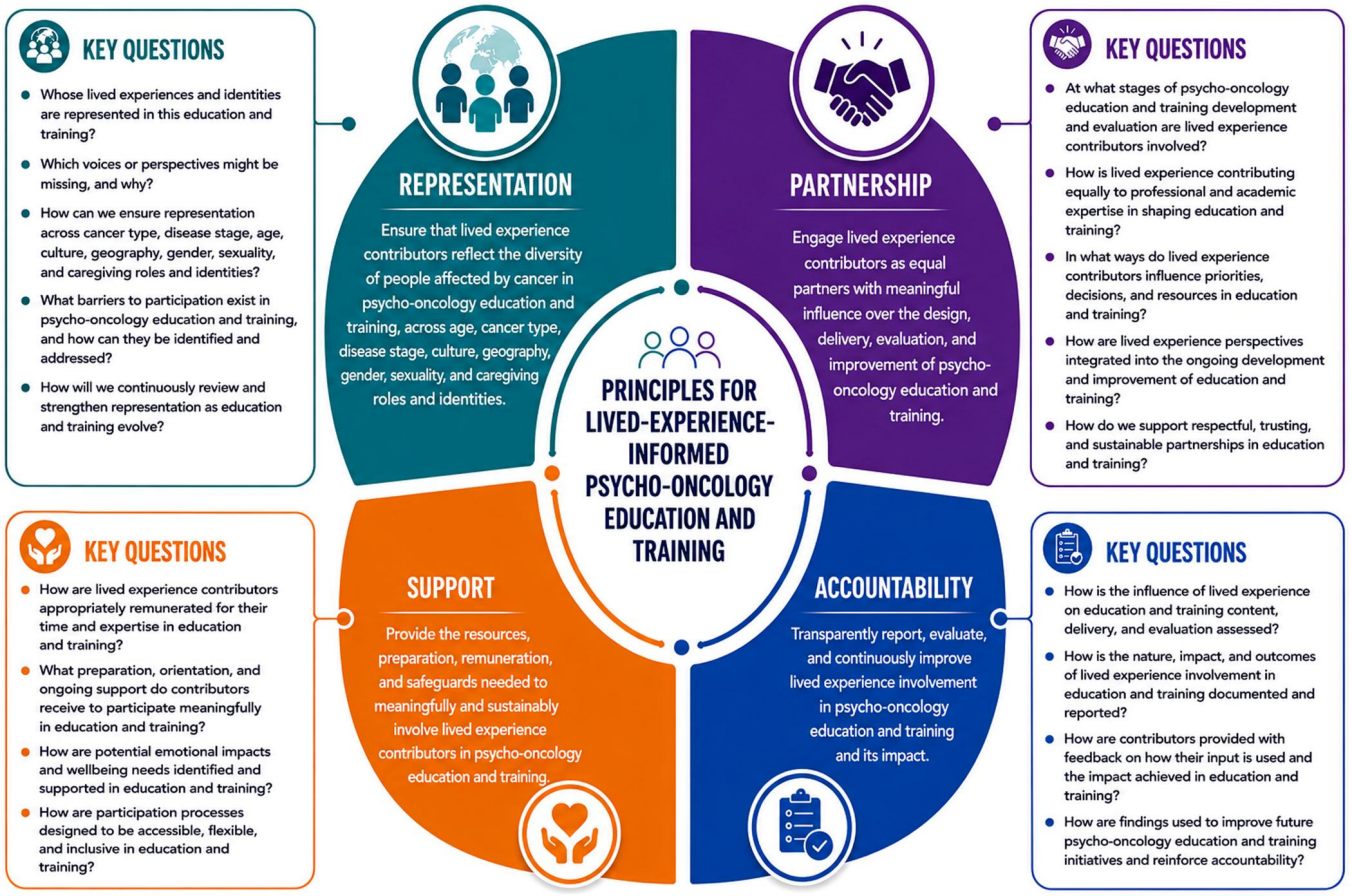
Principles for Lived Experience‐Informed Psycho‐Oncology Education and Training. Four principles for embedding lived experience expertise in psycho‐oncology education and training: representation, partnership, support, and accountability. Accompanying questions are designed to help educators integrate lived experience across design, delivery, evaluation, and continuous improvement of psycho‐oncology education and training.

### 5.1 Representation

Psycho‐oncology education and training programmes should actively seek diverse lived experience perspectives reflecting the populations that clinicians serve. Representation extends beyond disease type or treatment pathway. Diversity in age, gender, culture, education, health literacy, socioeconomic status, geographical location, family roles, sexuality, disability status, stage of disease, and survivorship trajectory shape psychosocial experiences. Educational programmes should therefore move beyond relying on a small number of familiar consumers and instead develop sustainable approaches for broad and inclusive engagement, such as targeted recruitment, partnerships with community organisations, flexible participation formats, reimbursement of costs, and tailored supports that reduce barriers to involvement. Diverse lived experience perspectives can also help clinicians appreciate that preferences for communication, decision‐making, support, and care may vary across individuals and sociocultural contexts. Representation is particularly important because consumer involvement initiatives can unintentionally privilege voices that are already more visible, resourced, or comfortable in academic and clinical environments [1, 5].

### 5.2 Partnership

Consumers should have meaningful opportunities to influence decision‐making throughout education and training planning, delivery, evaluation, and continuous curriculum improvement. Partnership involves shared authority regarding learning priorities, educational content, teaching methods, education and training outcomes, and evaluation processes. Rather than asking consumers to endorse predetermined education and training agendas, educational teams should involve lived experience contributors from the earliest stages of curriculum development. The movement from learning about consumers to learning with consumers is central to contemporary partnership in health professional education and training [1, 4]. Partnership also means recognising that different forms of expertise are complementary. Clinical expertise and experiential expertise serve distinct but equally important functions in developing high‐quality education and training.

### 5.3 Support

Meaningful consumer involvement requires adequate resources. Consumers should be appropriately remunerated for their contributions, provided with sufficient preparation and orientation, and offered flexibility to accommodate health, caregiving, employment, or accessibility needs. Support should also recognise that contributors have different preferences, capacities, and ways of engaging. Emotional wellbeing must also be prioritised. Education and training programmes should consider mechanisms for debriefing, support, and withdrawal from activities when participation becomes burdensome or distressing. Support should not be viewed as an optional enhancement; it is fundamental to ethical and sustainable partnership. Recent synthesis of consumer partnership in health professional education emphasises that meaningful and inclusive partnerships require relational and structural support, including time, resources, and attention to emotional labour [1].

### 5.4 Accountability

Education organisations and providers should be transparent about how lived experience informed each offering. Reporting should describe who was involved, how involvement occurred, what influence consumers had on decision‐making and what impacts were observed. The GRIPP2 reporting checklists provide useful guides for improving transparency and consistency in reporting consumer and public involvement activities [3]. Beyond reporting processes, accountability requires evaluation and ongoing development. Education and training programmes should examine whether and how lived experience involvement improves educational relevance, clinician competencies, learner attitudes, communication practices, and ultimately consumer experiences of care.

## 6 From Optional to Required

Across healthcare, consumers are increasingly recognised as partners in the design, delivery, evaluation, and improvement of services. Similar shifts are occurring in education. Recent scholarship increasingly emphasises that consumers should contribute not only to training activities but also to curriculum development itself [1, 4]. Such approaches demonstrate lived experience involvement can be embedded into educational planning rather than added at the point of delivery. Psycho‐oncology should not lag behind these developments. In many respects, the field is uniquely positioned to lead them. The discipline already recognises the importance of subjective experience, person‐centred communication, and collaborative care. Integrating lived experience into workforce development therefore represents a logical extension of existing values rather than a radical departure from current practice.

Importantly, this shift does not imply that every education and training session requires consumer co‐facilitation or that educational decisions should be driven solely by consumer perspectives. Rather, it acknowledges that experiential expertise provides essential knowledge that cannot be obtained through professional education and training alone. Lived experience contributors need not replace clinicians, educators, or researchers. Instead, they should join them as equal partners in shaping the future psycho‐oncology workforce.

## 7 Future Directions

Psycho‐oncology is well positioned to establish clear expectations for lived experience involvement in education and training. Professional societies, educational institutions, cancer centres, and education and training organisations can all contribute to this agenda. Several practical actions could accelerate progress. First, organisations should routinely report lived experience involvement in psycho‐oncology education and training initiatives. Transparency creates accountability and allows successful models to be shared and adapted. The GRIPP2 checklists offer an established guide for improving the reporting of consumer and public involvement, including the nature, extent, and impact of involvement [3]. Meaningful involvement is not always straightforward and may require additional time, resources, training, and relationship‐building, particularly when working to engage communities that have been historically underrepresented in healthcare and research. Second, expectations for consumer partnership could be incorporated into education and training standards, accreditation processes, grant schemes, and workforce development frameworks. This would shift lived experience involvement from a desirable addition to an expected feature of high‐quality psycho‐oncology education and training. Third, research is needed to evaluate the impact of lived experience‐informed education and training on educational outcomes, clinician behaviour, service delivery, and consumer experiences. While the theoretical rationale, and emerging evidence, is compelling, stronger empirical evidence will help guide implementation and resource allocation. Future work should also examine how partnerships with lived experience contributors can be sustained beyond individual courses or projects [1]. Finally, psycho‐oncology must continue to broaden whose voices are heard. Genuine person‐centred care requires attention to diversity, equity, inclusion, and cultural safety in all aspects of workforce development.

## 8 Conclusion

Psycho‐oncology has long championed person‐centred care and recognised the importance of understanding the lived experience of cancer. Yet, the education and training of the psycho‐oncology workforce has not consistently reflected these principles. Lived experience involvement in clinician education and training should no longer be regarded as an optional addition or educational enhancement. It should be recognised as a fundamental source of expertise that enriches curriculum design, strengthens workforce development, and helps align education and training with the realities of living with and beyond cancer [1, 4]. Achieving this vision requires genuine partnership built on representation, support, and accountability. By embedding lived experience throughout the development, delivery,evaluation, and continuous improvement of psycho‐oncology education and training we can better prepare clinicians to meet the psychosocial needs of people affected by cancer.

## Lived Experience or Public Contribution

This viewpoint was co‐developed by educators, researchers, clinicians, and a with lived experience expert. Co‐first author Sue Woodall contributed lived experience expertise throughout the conception, development, and refinement of the manuscript. The paper is also informed by the authors' collective experience undertaking consumer partnership and co‐design initiatives in psycho‐oncology education and training. Lived experience helped shape the paper's key arguments, the proposed principles and recommendations.

## Author Contributions

D.H. and S.W conceived the paper and wrote the first draft. D.H., S.W., A.H., J.C. E.D., A.S. and N.H.H. provided revisions and approved the final version.

## Funding

The authors have nothing to report.

## Conflicts of Interest

The authors declare no conflicts of interest.

## Data Availability

Data sharing is not applicable to this article as no datasets were generated or analysed during the current study.
